# Linguistic typology of motion events in visual narratives

**DOI:** 10.1515/cogsem-2022-2013

**Published:** 2022-10-17

**Authors:** Irmak Hacımusaoğlu, Neil Cohn

**Affiliations:** Department of Communication and Cognition, Tilburg School of Humanities and Digital Sciences, Tilburg University, Tilburg, The Netherlands

**Keywords:** comics, linguistic relativity, linguistic typology, motion events, visual language

## Abstract

Languages use different strategies to encode motion. Some use particles or “satellites” to describe a path of motion (Satellite-framed or S-languages like English), while others typically use the main verb to convey the path information (Verb-framed or V-languages like French). We here ask: might this linguistic variation lead to differences in the way paths are depicted in visual narratives like comics? We analyzed a corpus of 85 comics originally created by speakers of S-languages (comics from the United States, China, Germany) and V-languages (France, Japan, Korea) for both their depictions of path segments (source, route, and goal) and the visual cues signaling these paths and manner information (e.g., motion lines and postures). Panels from S-languages depicted more path segments overall, especially routes, than those from V-languages, but panels from V-languages more often isolated path segments into their own panels. Additionally, comics from S-languages depicted more motion cues than those from V-languages, and this linguistic typology also interacted with panel framing. Despite these differences across typological groups, analysis of individual countries’ comics showed more nuanced variation than a simple S–V dichotomy. These findings suggest a possible influence of spoken language structure on depicting motion events in visual narratives and their sequencing.

## 1 Introduction

Languages differ in how they might describe the same situation, but typically use a limited set of structural options. One difference identified across languages is the pattern for encoding the path of motion events, i.e., the direction of the movement of an object relative to the ground or another object (Slobin 2004; Talmy 1985)*.* Similarly, sequencing in visual narratives, like comics, also differs in conveying situations with motion events (Cohn 2013; McCloud 1993). Thus, we ask whether the cultural systems of visual narratives differ in their paths and whether the depictions of paths are affected by the languages of their authors.

### 1.1 Paths in spoken languages

**Figure 1:**
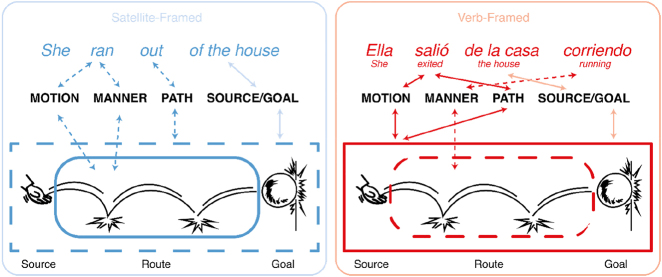
The lexicalization pattern of motion events in Satellite-framed (S) versus Verb-framed (V) languages and hypothesized mapping to a visual representation. Solid lines depict how the main verb would correspond to a visual depiction, while dashed lines indicate other aspects of path constructions.

The path-of-motion can be segmented into different components: source (i.e., starting point of an object along the path), route (i.e., the traversal of the path itself), and goal (i.e., the endpoint of an object along the path). The object moves from the source to the goal and passes along a route (Figure 1). These semantic components give the path information while manner characterizes the action or *how* the object moves (e.g., *running, crawling, climbin*g).

These semantic components vary in how they manifest in different languages. Talmy’s (1985) classic typology posits that languages use two different lexicalization patterns to map the path-of-motion. In languages like English, German, and Dutch, manner is typically encoded in the main verb while the path information is encoded through additional particles or “satellites” (e.g., prepositions) associated with the verbs. These languages are thus called “Satellite-framed” or “S-languages.” Thus, path changes can be conveyed easily by adding prepositions while the main verb slot is available for the manner information, as shown in Figure 1.

In contrast, languages like Turkish, French, and Japanese typically use the main verb (e.g., *to enter, to pass, to exit*) to encode the path information. They are thus called “Verb-framed” or “V-languages.” To express the manner information, V-languages then require additional phrases or subordinate clauses (e.g., Spanish *en courant*, ‘*by running’*). Manner is expressed outside the main verb since the path verb occupies the main verb slot, as in Figure 1. Then, the attachment of manner phrases must occur for all path segments (e.g., for source, goal, or route individually); otherwise, the manner information (*running*) would only apply to the path segment it is used for.

However, lexicalization patterns of motion events might differ based on the type of motion, instead of applying to all motion events equally (Özçalışkan 2015). The key is whether the motion involves crossing a spatial boundary, which refers to motion *into*, *out of,* or *over* a bounded region. Manner verbs can appear in V-languages if there is no traversal of a spatial boundary e.g., *She ran toward the house*. With a bounded region such as going into a new place, then V-language speakers need path verbs for the change of location e.g., *She entered the house by running* as opposed to *She ran into the house* (Aske 1989; Özçalışkan 2015; Slobin and Hoiting 1994).

Questions also persist whether *all* languages could be classified within the binary of S- or V-languages. Slobin (2004) proposed a third group of equipollently-framed languages, such as Chinese, which express path and manner equivalently through serial verb constructions. More recent works have shown variations in motion event constructions within groups and even within specific languages (e.g., Naidu et al. 2018). These and other observations led to proposals for classifying the construction types rather than classifying whole languages (e.g., Fortis and Vittrant 2016). For instance, Zlatev et al. (2021) showed that grouping languages based on constructions yielded four clusters, but groupings changed based on the comparison criteria. Thus, different clusters could potentially arise from comparing different constructions.

Within the S-/V-language dichotomy, motion event lexicalization patterns have been shown to place different costs on comprehenders. The auxiliary verb leads speakers of V-languages to tend to leave out manner, whereas in S-languages, the manner information is highly accessible, and actions become more salient compared to V-languages (Slobin 2003). In line with this, Berman and Slobin (1994) showed cross-cultural variation when children described a pictorial storybook. Children who spoke V-languages described the visuals by using path verbs and expressed manner only if necessary. In contrast, children who spoke S-languages used more diverse and fine-grained manner verbs. S-language speakers also described the manner-of-motion in their mental imagery more vividly than V-language speakers, who habitually described the physical and emotional setting (Slobin 2003). Similarly, novels from S-language writers use more detailed manner of actions compared to novels written by V-language writers, and translations of narrative texts from S-languages (English) to V-languages (Spanish) have been shown to replace manner with path verbs or omit manner entirely (Slobin 2000).

Moreover, Kita and Özyürek (2003) demonstrated cross-linguistic variation in gestural representations of motion events. They found that S-language speakers were inclined toward using one gesture that conflates both manner and path information, whereas V-language speakers preferably used separate gestures for each component. Thus, parallels arise between the way information is packaged in languages and the gestures of those speakers (see Özyürek et al. 2005 for replications with different motion events).

These findings imply effects of linguistic typology on comprehension and interactions with other domains. Thus, our primary question is whether these typological differences in spoken languages also influence the representation of motion events that people draw. For convenience, we here retain the original S/V typological classification of languages while acknowledging that more nuances may arise within languages on a constructional basis. To forecast our questions below, we wonder how paths in visual narratives within our corpus would relate to the original S/V classification and how they would deviate from it.

### 1.2 Paths in visual narratives

Visual narratives like comics provide a particularly compelling place to look at paths, since they both show motion events in single images (within a panel) and extend across sequences. In addition, Visual Language Theory (Cohn 2013) has argued for parallels in the structure between spoken languages and graphic communication. This theory proposes that, just as spoken or signed languages are fundamental human abilities to express meaning produced in a spoken or bodily modality and governed by a system of combinatorial principles, structured sequential drawings use similar principles in the mapping of graphics and meaning. Conventionalized systems of drawing can thus be deemed “visual languages,” which vary across cultures using shared structures among a particular group, similarly to the cross-cultural diversity of spoken languages. Highly codified visual languages appear in comics of the world, which often use systematically consistent graphics and patterned methods of narrative sequencing, and which then combine with written language (Cohn 2013; McCloud 1993)*.* Thus, Japanese manga uses a “Japanese Visual Language” that systematically differs from the “American Visual Language” used in comics from the United States (Cohn 2013, 2020). To what extent cross-cultural systems vary and overlap is an ongoing topic of analysis.

**Figure 2:**
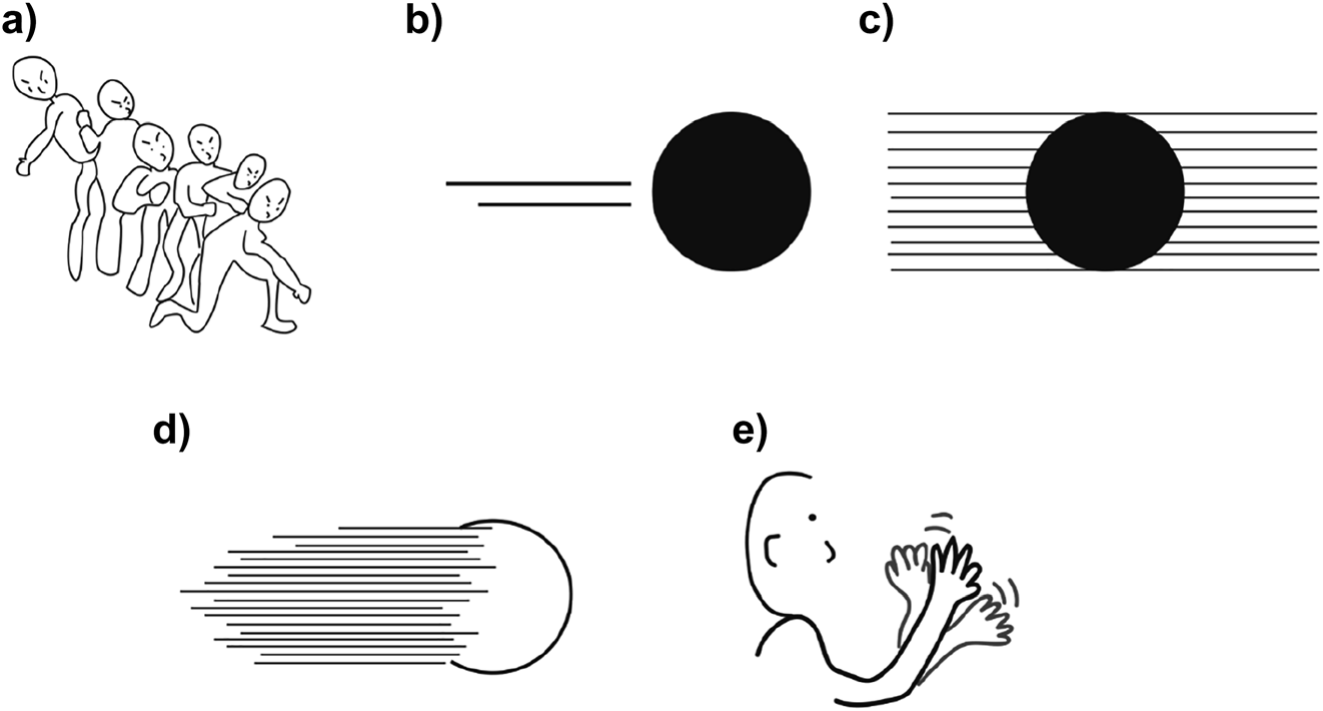
Visual cues i.e., a) postures, b) affixes, c) backfixing lines, d) suppletion, and e) repetition that are used to convey motion.

As in the challenge of conveying motion events in spoken languages, several strategies are used to depict dynamic events in two-dimensional static images (Cutting 2002). For instance, visual depictions show motion through the postures that figures take in actions (Figure 2a), and static representations implying motion are suggested to be processed similarly to dynamic events (Kourtzi and Kanwisher 2000). A figure’s depicted posture can create a prediction of the figure’s upcoming action even in static images (Kawabe and Miura 2006). This knowledge of visual postural depictions aids the comprehension of motion, even at younger ages (Friedman and Stevenson 1975).

Beyond postural cues implying motion events, graphics also depict paths directly using motion lines (also called speed lines or action lines) where a line trails behind a moving object to show the path it has traversed (Cohn 2013; McCloud 1993) (Figure 2b). Like affixes in languages, motion lines cannot stand alone and thus must be attached to their stems i.e., the moving object (Cohn 2013).

**Figure 3:**
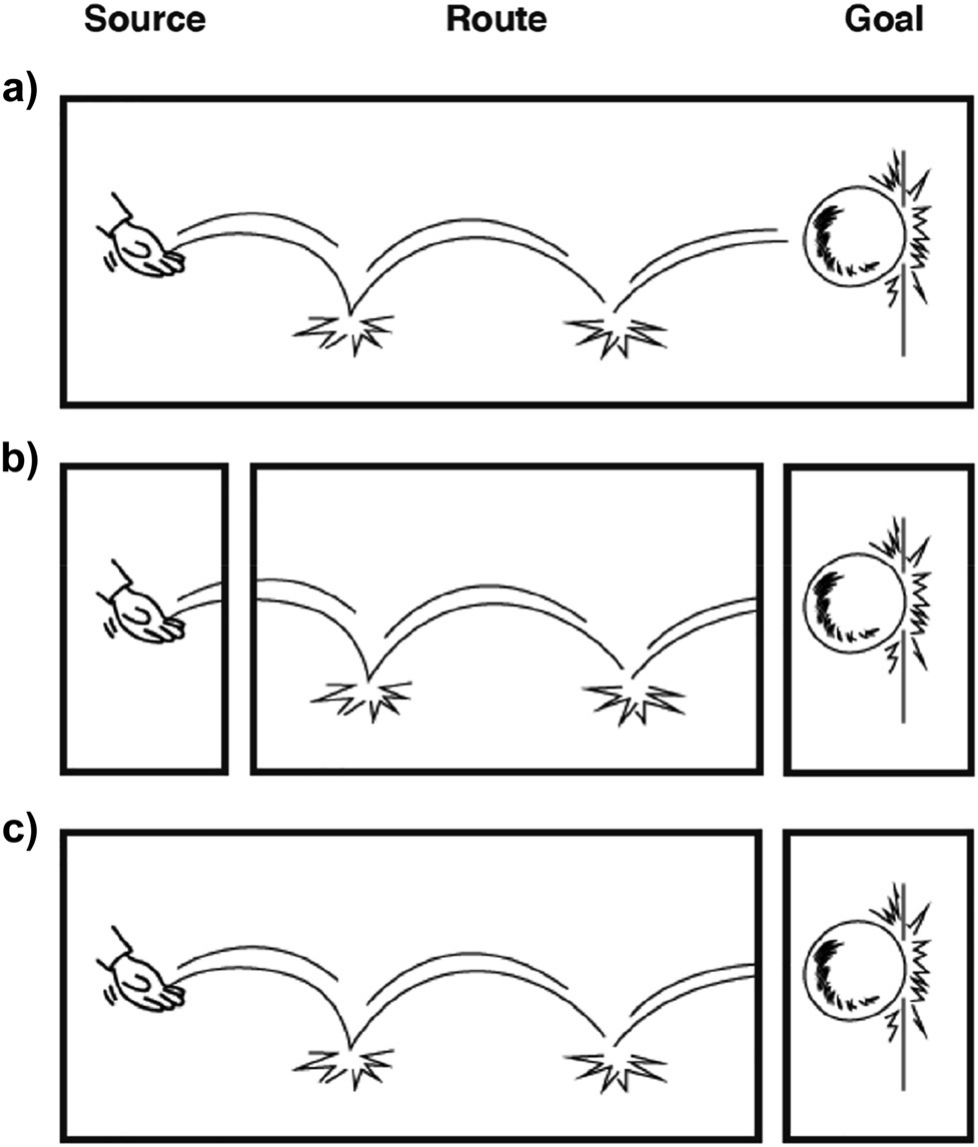
a) All segments (Source, Route, and Goal) of a path in a single panel. b) Path segments isolated to their own panels or c) an example of binary combinations. The curved lines show the route and manner of bouncing.

Across motion lines, the source of the path information appears at the starting point, and the goal at the endpoint (Figure 3a). In that sense, motion lines can directly indicate the traversal, making it possible to depict a change of location over a spatial boundary, unlike postural cues. Also, manner of the visual path is usually encoded through the shape of the middle part of the line, the route. For example, as in Figure 3, a bouncing object uses curved lines with midpoints along the route, implying the previous states where the ball impacted the ground. Spinning objects then might show a spiraling line. Also, all three path segments can be drawn at once, as in Figure 3a. Alternatively, each path segment could be isolated to its own panels (Figure 3b) or use binary combinations (e.g., Source – Route as in Figure 3c or Route – Goal).

Motion lines can imply the speed of a moving figure (Carello et al. 1986; Cutting 2002), by modulating the length and number of the lines (Hayashi et al. 2012), again reflecting the manner information. Also, several motion lines can attach to the background (i.e., backfixing lines; Figure 2c), which gives a sense that the viewer moves with the object, while the background is “blurred” (Ito et al. 2010; McCloud 1993). In contrast, certain parts of an object can be replaced with motion lines (i.e., suppletion; Figure 2d), making it seem like the object itself is blurred through its motion (Cohn 2013). Finally, parts of a figure (e.g., hands) can be repeated to demonstrate the motion (e.g., depicting multiple hands to give the sense of waving, as in Figure 2e). While backfixing lines, suppletion, and repetition all convey motion, in these cases, path information remains more implicit in contrast to the overt paths conveyed by motion lines.

Because motion lines directly show the path of a motion event, they may aid the comprehension of depicted motion in single images (Brooks 1977; Friedman and Stevenson 1975). Motion lines help people better understand the path-of-motion and remember the direction of a moving object compared to the objects depicted without lines (e.g., Geisler 1999; Kawabe and Miura 2006). Also, the presence of lines, including backfixing lines, depicts motion better than both no lines and lines moving in the wrong direction (Ito et al. 2010). Further, Cohn and Maher (2015) found shorter self-paced viewing times for comic panels with normal motion lines than no lines, and even longer times to reversed lines. Yet, neural responses to scenes where motion lines were erased were found to activate comparable processes as reversed lines, suggesting that the motion lines were helpful beyond having no lines. This effect was modulated by participants’ comic reading expertise, supporting the notion that motion lines are conventionalized in a “visual vocabulary”.

Unlike postural cues, the comprehension of motion lines develops as children age. Younger children can have trouble comprehending motion lines and only interpret them as symbolic representations as they age, at which point they rely on motion lines more than postural cues (Friedman and Stevenson 1975). However, postures and motion lines together contribute to comprehension better than each does alone (Kawabe and Miura 2006). Furthermore, similar to variations across languages, the representations of motion lines vary cross-culturally, such as Japanese manga and American comics using different depictions of motion (Cohn 2013; McCloud 1996, 1993). Finally, people who are not exposed to motion lines in their cultures have difficulty comprehending that they depict motion (e.g., Kennedy and Ross 1975), and their understanding is further modulated by experience (Cohn and Maher 2015).

Cross-cultural research in visual narratives has also demonstrated differences in the framing structure of panels (Cohn 2013, 2020). Because panels of comics act as “attentional units”, highlighting different aspects of a scene, panels can be categorized based on the number of primary characters they depict (Cohn 2013). Panels are categorized as Macros if they depict multiple active entities, Monos if depicting a single active entity, Micros if showing less than one active entity, and Amorphic panels if depicting no active entities. Since scenes can be framed in these different ways, this could affect how motion events are segmented. Prior work has found that American and European comics use Macros more than any other panel type. However, Asian comics typically use as many if not more Monos than Macros, and greater frequencies of Micros, reinforcing a focus on certain parts rather than full scenes (Cohn 2013, 2020). Nevertheless, these framing-type tendencies cut across the linguistic typology of motion events, such as French (a V-language) and German (an S-language) comics, which share a similar framing structure. Thus, it remains an open question whether attentional framing might affect how motion events are represented.

To summarize, various graphic devices act as visual cues to convey motion events, implying path and manner information. However, whether these cues might be influenced by how paths are encoded in their drawers’ native languages remains unknown. Given that both verbal and visual representational systems express paths, might the typological distinctions of S- and V-languages constrain how motion events and paths manifest in visual narratives?

As described above, studies of verbalized motion events have often relied on visual narratives as elicitation stimuli. For example, the pictorial narratives of *Frog, where are you?* (Mayer 1969) have been used in tasks with participants from various cultures to verbalize what they see in the given visual narratives (e.g., Berman and Slobin 1994; Naidu et al. 2018; Slobin 2000). Nevertheless, in using visual narratives as stimuli, the structures of the depicted paths could affect elicitation results if that book depicted paths in a particular way that is (in)consistent with a given linguistic typology. Such variation potentially originating from the visuals themselves has not been considered in this research.

In addition, visual narratives have been used as a context to investigate linguistic typological differences. Molés-Cases (2020a, 2020b) conducted a multilingual corpus study on the translation of manner in the text of comics. Here, manner was usually omitted in translations from S-languages to V-languages, but the omission of manner in the text was sometimes compensated through visual codes (i.e., drawing the manner information). Thus, the manner information, omitted in the text, is not lost in the translation through the aid of the visual language. This corpus study showed evidence for the multimodal role of graphics in minimizing the consequences of the effect of language typology on translations.

While these works have used visual narratives to study typological differences in S- versus V-languages, they do not address how this information is encoded in the visual narratives *themselves*, rather than how people verbalize what they see in those depictions or structure the accompanying text. To this end, Tversky and Chow (2017) carried out the first analysis of motion event typologies in visual narratives by focusing on the depiction of action in panels from S- versus V-languages comics. They asked native English (S-language) and Japanese (V-language) speakers to rate panels along a scale depicting action versus scene-setting in S-language comics (English and Mandarin) and V-language comics (Japanese and Italian). Higher action ratings were given to comics in S-languages than in V-languages. Their results suggested an effect of language typology on the depictions and salience of actions in visual narratives.

Instead of using action-ratings as a proxy for motion events and paths, here we look at path segments directly in 85 comics and examine cues (i.e., affixes, postures, suppletion, backfixing lines, and repetition) signaling path- and manner-of-motion. We also consider the attentional framing structure of the comics’ panels. We hypothesized that overall, comics from S-languages would depict more path segments than comics from V-languages based on findings that S-language speakers tend to mention more path segments than V-language speakers (Slobin 2005). The saliency found in S-languages of encoding both the motion and manner in the main verb should be especially salient in routes, where manner is typically represented (as diagrammed in Figure 1). In contrast, V-languages focus their main verb on motion and path information, so they should have less focus overall on routes (Figure 1). Thus, we further expected a greater proportion of routes in S- than V-languages. However, since V-languages isolate manner into a second verb, we also predicted that when routes are shown, they may be segmented into their own panels more often in V-languages than in S-languages (as in Figure 3b). Finally, because sources and goals are equally salient across language typologies, we hypothesized that there would be no difference in the representation of sources or goals across comics.

We also hypothesized differences in the visual devices signaling paths, or “path cues.” Since motion events have been suggested to be more salient in S-languages than in V-languages (Slobin 2003), we predicted that more cues would appear in S-language comics than V-language comics. Specifically, motion lines are expected to be used more often in S-languages since routes, and manner, are typically conveyed through motion lines. Yet, since manner can be omitted in V-languages—or isolated in a secondary verb—V-language comics are not expected to rely on motion lines as much. Instead, we predicted that V-language comics would depict postures more often than affixes of motion lines because postures imply motion without showing boundary-crossing directly and leave out fine-grained manner information made overt by affixes.

Finally, we expected a relationship between attentional framing structure (i.e., segmentation of panels) and the routes. Because panels’ framing can vary across cultures, this could affect how motion events are represented across panels. Specifically, because V-languages separate manner from motion, we predicted V-language comics might use more segmented scenes (such as with a focus on Mono panels, as in Figure 3b) than S-languages where the motion and manner are kept together (such as with a focus on scene-level depictions in Macro panels, as in Figure 3a). Given the relationship between manner and routes, we predicted that routes would correlate with a trade-off between Macros (S-languages) and Monos (V-languages).

## 2 Method

### 2.1 Materials

We selected 85 comics annotated within the Visual Language Research Corpus (VLRC; Cohn et al. In Prep) for aspects of motion events and their visual cues. More information regarding the corpus and its data can be found at http://www.visuallanguagelab.com/vlrc. These comics were representative of books from S-languages (China, Germany, the United States) and V-languages (France, Japan, Korea), as listed in Table 1. Within our selection from the United States, our analysis focused on mainstream comics (i.e., the superhero genre) and US manga or Original English Language (OEL) manga, which are works written by English speakers but drawn in Japanese manga style (i.e., using the Japanese Visual Language). Prior corpus analysis has suggested that the storytelling of US manga is balanced between patterns resembling structures from US mainstream comics, with which they share a country and market, and Japanese manga, which their visual style is imitative of or influenced by (Cohn 2020). These books thus provide an interesting place to analyze the crossing of authors who speak an S-language (English) but draw with Japanese Visual Language, which originally aligned with a V-language (Japanese).

**Table 1:** Types and origins of comics included in our sample from the Visual Language Research Corpus.

Comic type	Country	Language	Language typology	Average pages	Total panels	Total books	Dates
Chinese Manhua	China	Mandarin	S-language	28	935	6	2002–2005
French Bande dessinée	France	French	V-language	20	2,343	10	1985–2013
German Comics	Germany	German	S-language	29	1,680	8	1987–2010
Japanese Manga	Japan	Japanese	V-language	19	2,898	15	2003–2013
Korean Manhwa	Korea	Korean	V-language	22	2,227	15	1987–2007
US Mainstream Comics	United States of America	English	S-language	21	2,873	15	2004–2014
US Manga	United States of America	English	S-language	24	3,348	16	1991–2006

### 2.2 Areas of analysis

We analyzed each book through two primary dimensions: path segments and path cues. Exploratory analyses also examined attentional framing structure. Four annotators blind to the hypotheses of the study independently annotated books across all areas of analysis. Coders completed coursework on Visual Language Theory along with training in the specific fields of annotation. All annotators had to reach a threshold of 80% agreement on practice books for annotations before annotating the actual corpus.

#### 2.2.1 Path segments

Path segments were coded for the number of sources (i.e., the starting point), routes (i.e., the midpoint or path itself), and goals (i.e., the endpoint) per panel. Panels could also have path segments isolated to their own panels (e.g., Source-only). We recorded the total path segments per panel and then divided this by the number of panels per book to derive the mean number of instances. Then, these means for each book were averaged across annotators for the final analyses.

#### 2.2.2 Path cues

The second area of analysis identified the visual cues implying the path- and manner-of- motion. These graphic devices included affixes, backfixing lines, postures, suppletion, and repetition, as in Figure 2. Prototypical motion lines were coded as affixes, while the lines set behind a figure in the background as backfixing lines. The postural cues were identified as the poses indicative of the character’s actions. The replacement of certain parts of a figure with motion lines was coded as suppletion and repetition of all or parts of a figure to convey motion as repetition. We recorded each instance of a visual cue, and the mean number of path cues was calculated in the same way as for path segments. We also computed the sum of all cues used in each panel and averaged them per book.

#### 2.2.3 Attentional framing structure

Finally, attentional framing structure of panels was annotated in line with previous research (Cohn 2013, 2020). Prior work (Cohn 2020) has identified one of the most salient aspects of characterizing framing of a comic as the ratio of panels showing scene level information (Macros, i.e., panels depicting multiple active entities) or individual characters (Monos, i.e., panels with one active entity). We thus characterized each book’s framing by subtracting the average of Mono panels for a given book from its average of Macro panels. This resulted in a continuum where higher scores indicated a comic with more Macros and lower (or negative) scores indicating more Monos. This relative score aimed to characterize the degree to which panels throughout a book may or may not segment scenes into parts, which might be relevant for how motion events would be depicted across panels.

### 2.3 Data analysis

Our main analyses looked at path segments (Source, Route, and Goal) and path cues (Affix, Backfixing lines, Posture, Suppletion, and Repetition). Path segments isolated to their own panels (Source-only, Route-only, and Goal-only) were also examined. We analyzed path segments by using Repeated Measures ANOVAs with three levels (i.e., Source, Route, and Goal) of within-group factors and typology (S- and V- languages) or country (US mainstream comics, US manga, China, Germany, France, Japan, and Korea) as the between-group factor. We compared the proportion of isolated paths to overall segments (i.e., Source-only %, Route-only %, and Goal-only %) between typologies through Independent Samples *t*-tests. Independent Samples *t*-test also compared the total number of path cues (collapsing all cues) per panel in S- versus V- languages comics. For further analyses, we only included the most prototypical cues, namely affixes and postures, because low frequencies were observed for backfixing lines (3%), suppletion (2%), and repetition (0.06%). Thus, Repeated Measures ANOVAs were conducted with main path cues (Affixes and Postures) as within-group factors and typology or country as the between-group factor, with pairwise comparisons in cases of significant main effects and interactions.

For additional relationships, we conducted a linear regression with affixes and postures as the predictors on the dependent variable of routes and a follow-up correlation analysis to examine the relationship between those two cues on the condition of routes. An Independent *t*-test and a One-way ANOVA compared the Macro-Mono ratio between language typologies and countries, respectively. Finally, through correlation analyses, we looked at the relationship between Macro–Mono difference and routes for S- and V-languages separately.

## 3 Results

### 3.1 Path segments

#### 3.1.1 Typology

**Figure 4:**
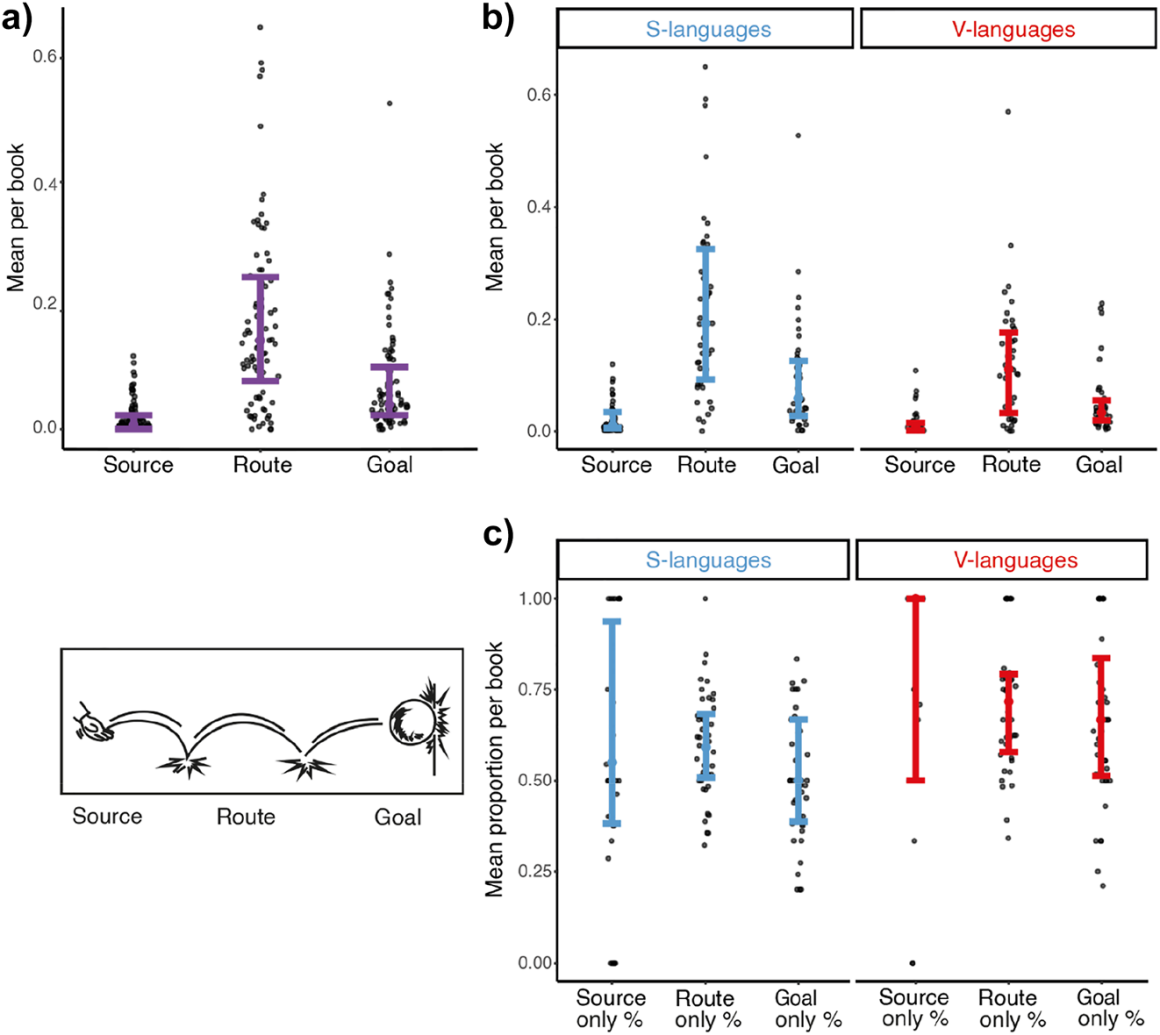
Path segments a) across all comics, b) averaged across typology, and c) for the proportion of path segments segmented into their own panel. Each dot represents a comic.

We began by investigating path segments (Source, Route, Goal) between language typologies (S- and V-languages). We found main effects of both path segments (*F* = 75.7, *p* < 0.001) and language typology (*F* = 11.4, *p* < 0.001), and an interaction between them (*F* = 5.7, *p* = 0.011). Overall, routes were depicted more often than goals and sources, while goals were used more than sources (all *t*s > 4.2, all *p*s < 0.001), as indicated in Figure 4a.

Comics from S-languages used more path segments than comics from V-languages (*t* = 3.3, *p* = 0.001), see Figure 4b. This difference primarily arose because S-language comics used more routes than V-language comics (*t* = 4.5, *p* < 0.001) but did not differ between their sources or goals (all *ps* > 0.2). However, within those path segments, V-language comics segmented routes isolated into their own panel at a greater proportion (M = 0.714) than S-language comics (M = 0.596) (*t* = 3.2, *p* = 0.002, as in Figure 4c), which also occurred for isolated sources and goals (all *t*s > 2.2, all *p*s < 0.035).

#### 3.1.2 Country

**Figure 5:**
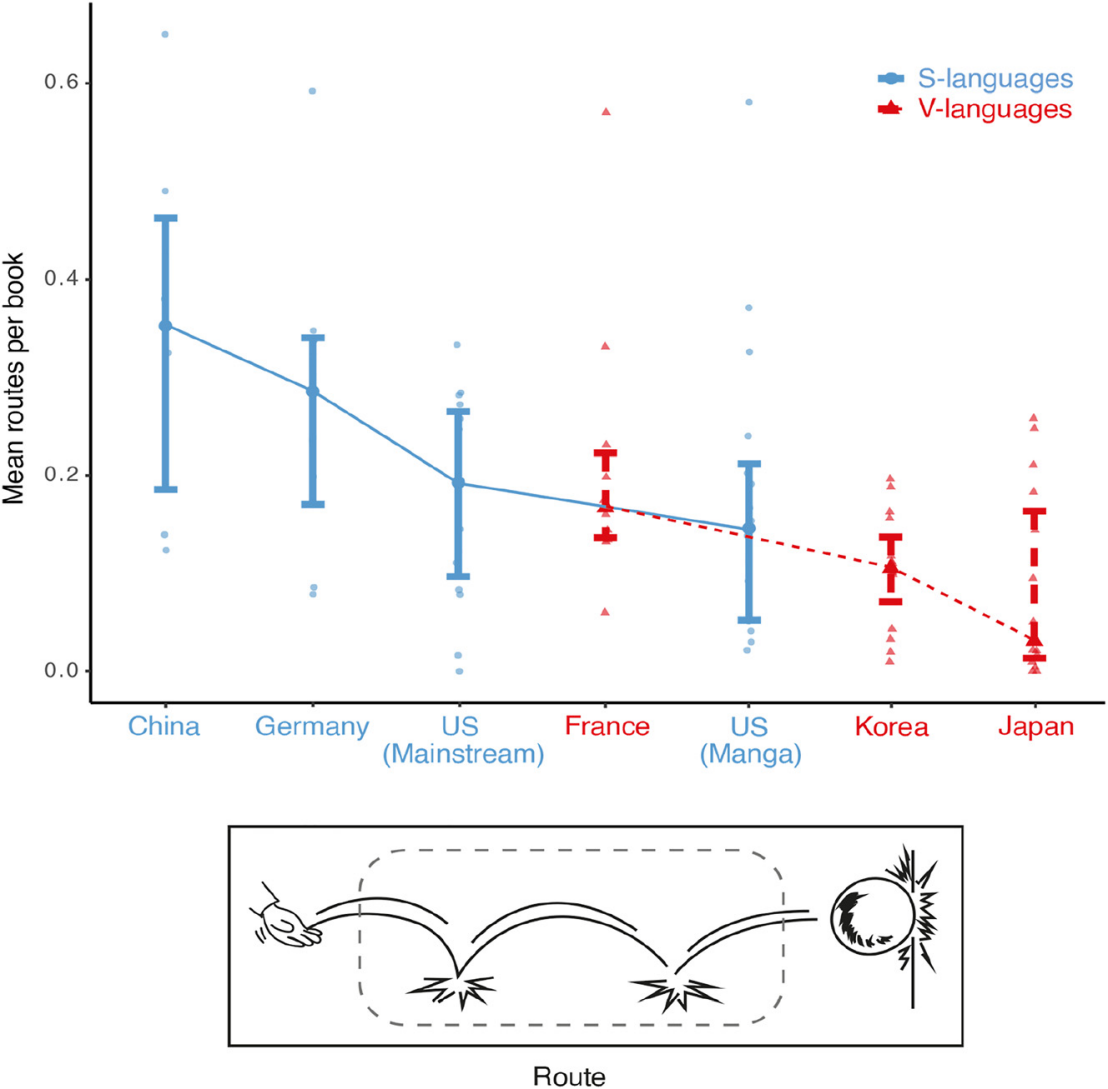
Routes averaged across countries. Each dot represents a comic.

We next examined path segments between countries. Main effects appeared for path segments (*F* = 100.5, *p* < 0.001), and country (*F* = 6.2, *p* < 0.001), and an interaction occurred between them (*F* = 3.5, *p* = 0.001). Path segments were used extensively in Chinese manhua than all other countries (all *t*s > 3.3, *p* ≤ 0.019) except for German comics (*t* = 1.4, *p* = 1.000). German comics had more path segments than all V-language comics (all *t*s > 3.4, all *p*s ≤ 0.016), except for French comics (*t* = 1.9, *p* = 0.589). Specifically, more routes were depicted in Chinese manhua than all other countries (all *t*s > 4.1, all *p*s ≤ 0.007) excluding German (*t* = 1.6, *p* = 1.000) and French comics (*t* = 3.1, *p* = 0.276), as in Figure 5. Routes were also depicted more in German comics than in Japanese manga (*t* = 5.06, *p* < 0.001) and Korean manhwa (*t* = 4.5, *p* = 0.001).

### 3.2 Path cues

#### 3.2.1 Typology

**Figure 6:**
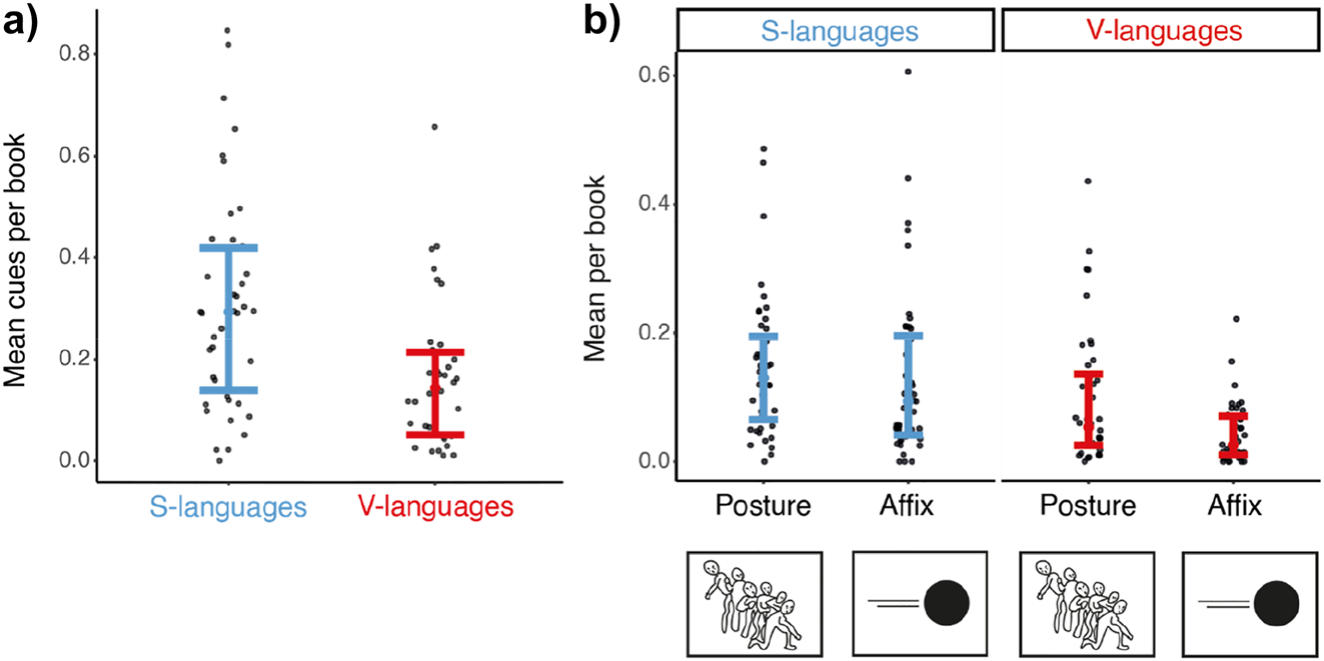
a) The mean number of cues (i.e., collapsing postures, affixes, backfixing lines, suppletion, and repetition) used per panel per book across typology b) affixes and postures averaged across typology. Each dot represents a comic.

We first examined the total number of path cues (collapsing Affix, Backfixing lines, Posture, Suppletion, and Repetition) across typology. Regardless of the cue type, comics produced by S-language speakers had more cues depicted per panel (*M* = 0.306, SD = 0.207) than comics from V-languages did (*M* = 0.161, SD = 0.139), *t* = 3.7, *p* < 0.001 (Figure 6a).

We then looked at affixes and postures specifically between language typologies. Main effects appeared for both path cues (*F* = 5.7, *p* = 0.019) and language typology (*F* = 14.5, *p* < 0.001). Cues were depicted more in S-language comics than in V-language comics (*t* = 3.8, *p* < 0.001), and overall postures were used more than affixes (*t* = 2.3, *p* = 0.019).

Post-hoc comparisons indicated that S-language comics used affixes (*t* = 3.7, *p* < 0.001) more than V-language comics while their postural cues did not vary (*t* = 2.2, *p* = 0.087), see Figure 6b. A trend appeared for using postures over affixes within V-languages (*t* = 2.4, *p* = 0.058), while within S-language ones, no difference arose between the two cues (*t* = 0.8, *p* = 0.402).

#### 3.2.2 Country

**Figure 7:**
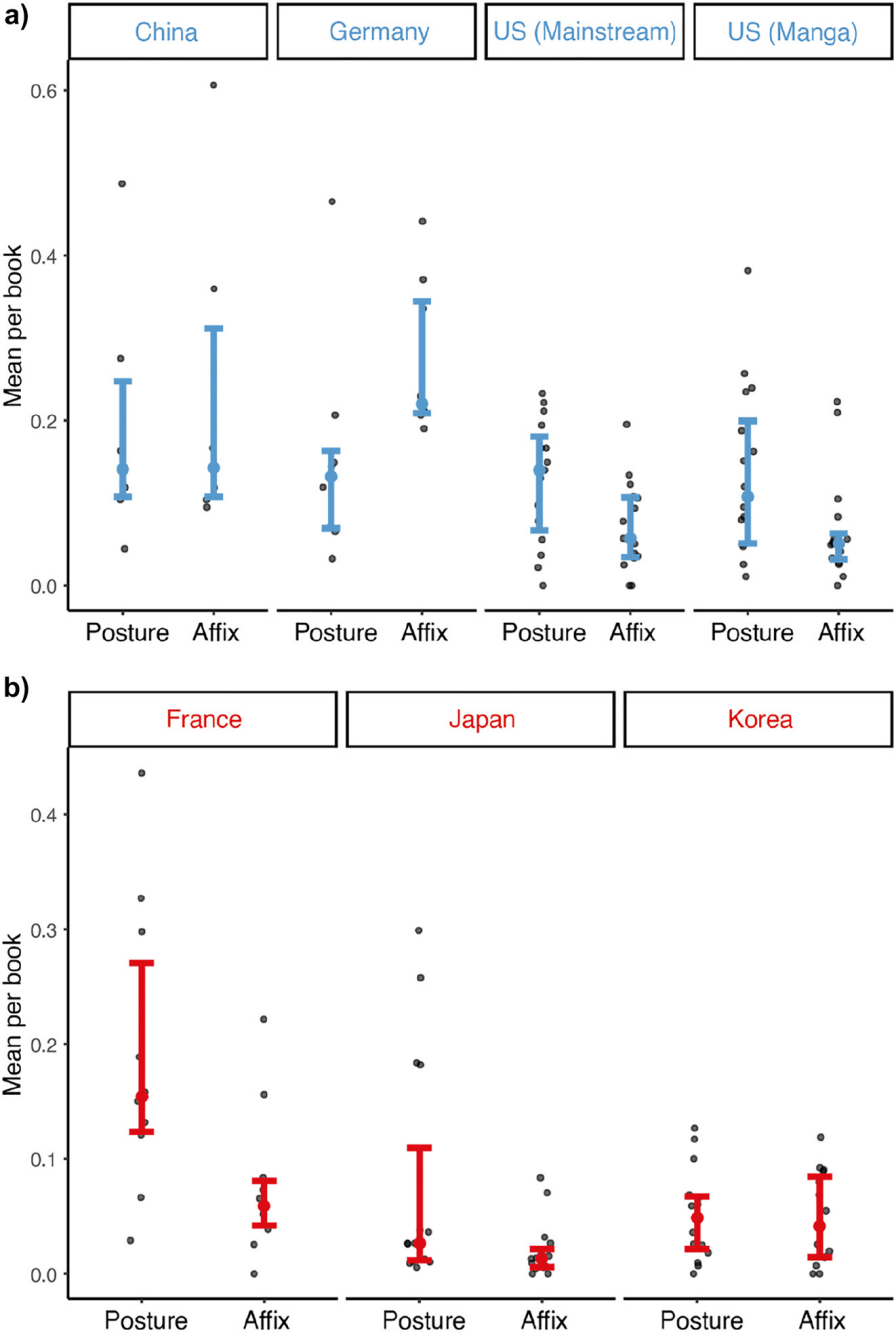
a) Main path cues (affixes and postures) averaged across countries of S-languages and b) V-languages. Each dot represents a comic.

We observed a main effect of country (*F* = 10.3, *p* < 0.001), and an interaction between path cues and the country (*F* = 2.07, *p* = 0.002). Similar to path segments, cues were depicted more often in Chinese manhua and German comics than in all other countries (all *t*s > 3.6, all *p*s < 0.007), excluding French comics (*p*s > 0.135). However, cues in French comics varied from Japanese manga (*t* = 3.09, *p* = 0.035) and Korean manhwa (*t* = 3.06, *p* = 0.036) although all are V-language comics. Post-hoc comparisons demonstrated Chinese manhua and German comics used affixes more than other countries (all *t*s > 3.5, all *p*s < 0.034), indicating their greater frequency of cues came from affixes (Figure 7a). However, only French comics and Korean manhwa differed from each other in postures (*t* = 3.8, *p* = 0.014), see Figure 7b.

### 3.3 Linear regressions and correlations

#### 3.3.1 Path cues and routes

We next conducted a linear regression with routes as the dependent variable and affixes and postures as predictors, resulting in a significant effect (*F* = 137.9, *p* < 0.001, *R*^2^ = 0.771). Both affixes (*t* = 10.3, *p* < 0.001) and postures (*t* = 9.4, *p* < 0.001) were found to be predictors of routes. Follow-up analysis showed a negative correlation between affixes and postures on routes. Although both cues predict routes, they do not tend to occur together on depictions of routes (*r* (83) = −0.414, *p* < 0.001). In other words, the more that affixes were used to depict routes, the fewer that postural cues appeared on those routes and vice versa.

#### 3.3.2 Attentional framing structure

**Figure 8:**
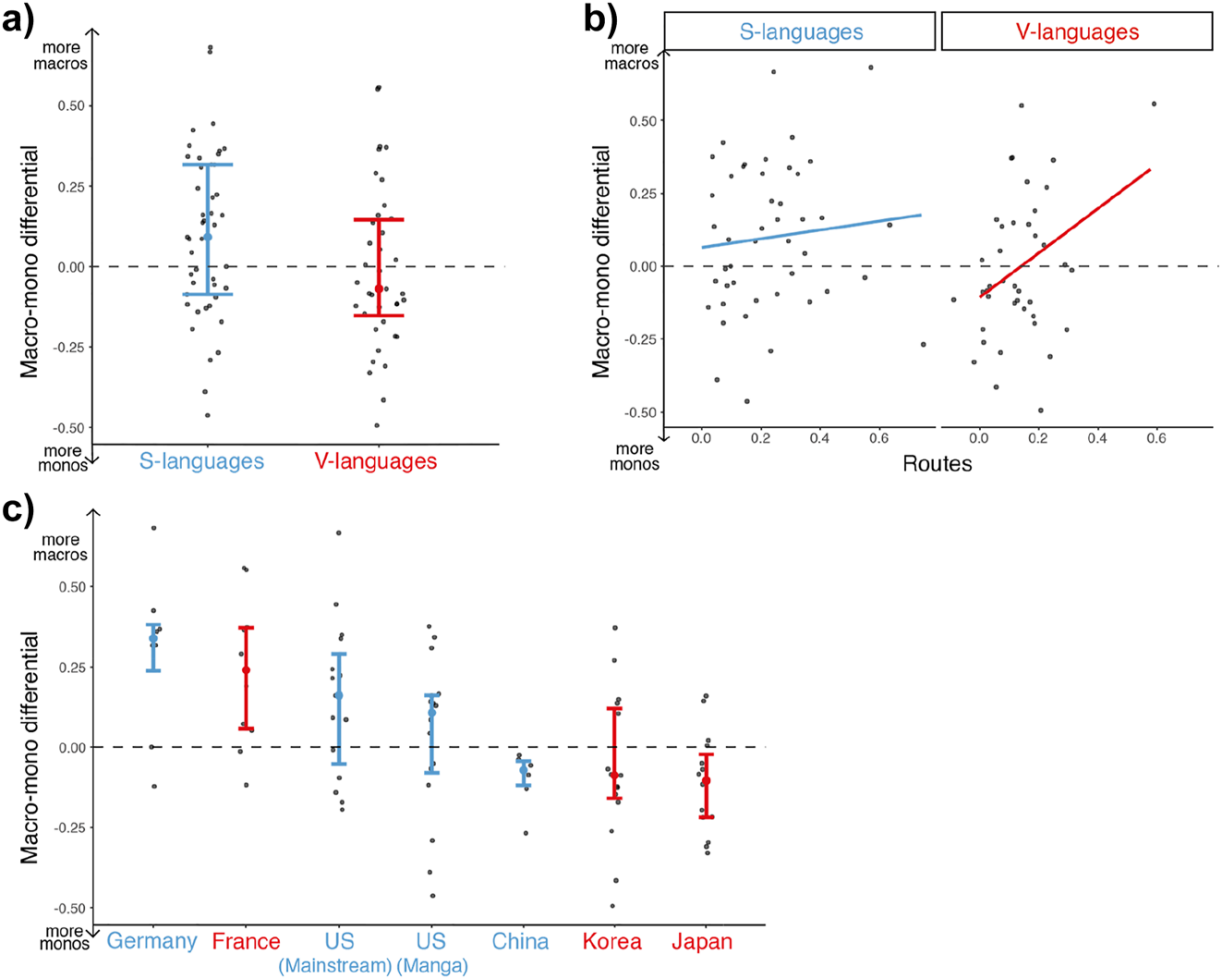
a) The difference between Macros and Monos averaged across typology. b) The relationship between this ratio and routes for S- and V-languages. c) Macro–Mono ratio averaged across countries. More positive numbers indicate more Macro panels, while more negative numbers indicate more Mono panels. Each dot represents a comic.

Our final analysis examined how motion events interacted with attentional framing structure (i.e., how much information is depicted in a single panel). We examined the ratio between Macro panels (i.e., framing multiple active entities) and Mono panels (i.e., framing a single active entity) across typologies (as in Figure 8a), and an effect near the threshold of significance appeared (*t* = 1.9, *p* = 0.056), implying that S-language comics tend to have more Macros (*M* = 0.099, SD = 0.257) while V-language comics have more Monos (*M* = −0.007, SD = 0.248).

We next looked at the relationship between framing and routes (Figure 8b) and observed a positive correlation (*r* (38) = 0.380, *p* = 0.016), suggesting that V-languages with more macros also depicted more routes. However, we found no relationship between the Macro–Mono ratio and routes for S-language comics (*p* = 0.303).

Macro–Mono ratios also differed between countries (*F* = 5.6, *p* < 0.001), as in Figure 8c. German comics had the largest skew towards Macros, which were greater than all Asian comics i.e., Chinese, Japanese, and Korean (all *t*s > 3.2, all *p*s ≤ 0.025). French comics were the next most Macro heavy, which varied from both Japanese manga (*t* = 3.7, *p* = 0.005) and Korean manhwa (*t* = 3.2, *p* < 0.027). Finally, US mainstream comics also differed from Japanese manga (*t* = 3.1, *p* = 0.033) while US manga did not (*t* = 1.7, *p* = 0.560).

## 4 Discussion

We asked whether the depiction of motion events in visual narratives varied based on authors’ spoken languages as S-languages (e.g., English) or V-languages (e.g., Japanese). We therefore examined path segments and path cues across 85 comics from around the world (English, German, Mandarin as S-languages; French, Japanese, Korean as V-languages). In line with our predictions, S-language comics depicted more routes than V-language comics, but routes in V-languages were more often segmented into their own panels than the routes in S-languages. In addition, more cues appeared in panels from S-language comics, primarily affixes signaling the routes and the manner information, compared to V-language comics. Finally, a relationship between attentional framing and routes within V-language comics further supported that V-languages align with greater segmentation of visual narratives in general, but when they do not, they depict more routes. We elaborate on these findings below.

We first focused on routes, as the locus of the motion and manner of a visualized path. Overall, the routes (i.e., encoding the path itself) were depicted more often than goals, and goals were used more than sources. In spoken languages, goals are more salient than their sources (e.g., Regier and Zheng 2007), which was also the case in our corpus. The importance of routes over goals might be peculiar to visual narrative systems, in which motion lines showing the traverse of an object are beneficial in comprehension of motion (Cohn and Maher 2015).

We next turned to whether linguistic typology varied this distribution of path segments. Since S-languages conflate both motion and manner into the main verb, we had predicted greater proportions of routes in comics from those languages, and indeed comics from S-languages used routes more often than comics from V-languages. Given that V-language speakers tend to leave out manner completely (Özçalışkan 2015; Slobin 2003), having fewer routes in comics from V-languages is also in line with the typological differences of their authors. S- and V-language comics did not differ in their sources or goals, consistent with how both typologies give salience to source and goal information. Nevertheless, we also expected more routes segmented into their own panel in V-language comics than S-language comics, in line with V-languages’ expression of manner in a secondary verb or through other constructions, like prepositional phrases or adverbial clauses. This also was the case. Altogether these results suggested that the salience of paths in visual narratives aligns with the expectations from linguistic typologies of motion events.

Greater proportions of isolated routes appeared in V- than S-language comics, as was predicted since manner is typically isolated in a secondary phrase in V-languages. However, greater proportions of isolated sources and goals also appeared for those comics, which was not predicted, despite the expected finding of no difference overall between sources and goals across typological groups. Perhaps V-language authors are inclined to isolate routes, which might lead them to isolate other path segments as well. Alternatively, depicting sources or goals alone would imply a path without showing a change of a state. As mentioned, it was proposed that V-language speakers had to use path verbs, especially in case of a change of state (Özçalışkan 2015; Slobin 2004); thus, segmenting those components might have been habituated and, in turn, reflected on the depictions of paths.

However, the properties of visual narratives themselves may also interact with how motion events are represented. In this regard, we had also considered the possible influence of attentional framing of visual narratives (i.e., how much information is depicted in a single panel) on the segmentation of motion events. We observed a trend of using more Macro panels (i.e., depicting multiple active entities in a single panel) over Mono panels (i.e., depicting one active entity in a single panel) in S-language comics and the reverse in V-language comics. Such framing arose from a Western-Asian division in visual narratives, which might have led to the isolated framing of sources and goals in V-languages. However, in V-language comics routes also correlated with Macros, which would not segment motion events into separate panels. Thus, for V-language authors, using Macro panels might have reinforced the depictions of overall routes, although they were also inclined to isolate those routes more often. Such findings may suggest constructional patterns for visually depicting motion events that cut across both path segments and attentional framing, potentially also with path cues, to which we now turn.

Overall, S-language comics used more path cues than V-language comics in their panels. In addition, greater amounts of motion lines in S-language comics supported our prediction that affixes would be more salient in S-language comics than in V-languages, as motion lines convey the routes and thus the manner information. However, both S- and V-languages used postures comparably. As expected, we observed a trend toward using postures over affixes in V-language comics, suggesting different strategies might be preferred to characterize paths with a possible influence of linguistic typology. Moreover, both postures and affixes predicted routes although they do not tend to be depicted together. This finding suggests a possible trade-off between these two cues signaling routes. It would also support the idea that motion events are emphasized by particular cues alone, such as motion lines, rather than being depicted together with other types of cues (Juricevic 2017).

This possible trade-off implies that V-language comics might use postures for cueing path information more indirectly than the overt paths of motion lines. This could then align with the notion that V-language speakers do not usually express the action saliently. Instead, they tend to mention the environmental setting, which might help paths to be inferred (Slobin 2005), or to mention a figure at two sides of a boundary (e.g., outside of the house and then inside of the house) implying the boundary (e.g., the moment of *going* ***into*** *the house*) has been crossed (Özçalışkan 2015).

For further nuance to these findings, we then looked at path segments and their cues broken down by their countries. In line with Chinese manhua and German comics having the most path segments, especially routes, they also used the most cues, particularly affixes conveying the manner information. Although differences along the linguistic classifications were supported, we also found intra-typological variations. For instance, US comics (both mainstream and manga) had fewer path segments, specifically routes, than Chinese manhua despite both being classified as S-languages. This is consistent with the findings of Tversky and Chow (2017) that action ratings for Chinese comic panels were higher than for English comic panels. These results could come from Mandarin itself, which was proposed as a third “equipollent” framing type (Slobin 2004) because it uses constructions in line with both S- and V-languages. Indeed here, Chinese manhua used equal amounts of postures and affixes, unlike the trade-offs in cues found in other countries.

In addition, US comics more resembled the V-language comics in our corpus, with affixes used less frequently in both types of US comics compared to other S-languages. These might be due to the influence of Japanese manga on US comics overall. In our corpus, the publication date of US mainstream comics was between 2004 and 2014 (see Table 1). Japanese manga’s influence on US comics started in the 1980s, including the production of US manga by English (S-language) speakers in manga style (Cohn 2020). Indeed, US manga were similar in routes compared to US mainstream comics. Also, in framing structure, lower rates of Macro panels occurred in both types of US comics (particularly US manga) than European comics, trending more like Japanese manga (Cohn 2013, 2020). Such influences of manga on US comics of all types may thus have affected their depiction of motion events.

Similar to US comics, French comics also had paths between S- and V-languages. Specifically, the comparable routes to S-language comics could relate to our finding suggesting a relationship between attentional framing structure and routes for V-language comics. The more Macro panels V-languages had, the more routes were depicted. French comics indeed had more Macro panels than Mono panels, similar to other European comics (Cohn 2013), and this might have allowed greater depictions of routes. While French comics also depicted more cues than other V-language comics, they were mostly postures, in line with predictions about how V-languages would depict paths. The trade-off between affixes and postures on routes could explain why French comics had comparable routes to S-languages (Chinese and German) despite not relying as much on motion lines.

The diversity of intra-typological differences in these visual narratives might also relate to recent views proposing construction-based classifications of motion events beyond the S- versus V-language dichotomy (Zlatev et al. 2021). As described above, Mandarin has been described as “equipollently-framed” using constructions across the S/V-language divide (Slobin 2004), in line with our findings of more routes and equal amounts of visual cues. In addition, Blomberg (2014) demonstrated that French speakers preferred manner verbs (e.g., French *sauter*, *‘jumping’*) in specific situations such as vertical motion events (e.g., *jumping down*) despite being a typical V-language in other dimensions. Analogously, when we looked at the segmentation of scenes, French clustered with German comics, despite differing in their path cues. Perhaps the typologies of visual narratives might similarly group based on constructions in their motion events, which would be consistent with the overall constructional framework of Visual Language Theory (Cohn 2013). Further research will thus need to investigate whether we can characterize such motion events constructions more specifically within visual narratives and whether they again might align with the constructions for motion events in spoken languages.

Altogether, these results provide evidence that the graphic depictions of paths in visual narratives align with those in spoken languages, possibly interacting with visual narrative specific properties. Given these differences across visual narratives, our results raise concerns with using pictorial story books as an elicitation tool to study spoken languages. Many psycholinguistic studies examining motion events have used visual narratives to sponsor verbalized descriptions and linked those findings to participants’ spoken languages. However, our findings show that visual narratives are not “neutral” in their structures, which indeed may differ from each other in systematic ways. Motion events in *Frog, where are you?* (Mayer 1969) might have influenced experimental findings, having been produced by a speaker of an S-language. Since visual structures might differ based on different cultures and languages, it is worth considering those structures in visual narratives themselves.

While we have found evidence for a relation between the linguistic typology of motion events and visual narratives in this corpus, our current annotations remain limited for characterizing such structures. For instance, our primary analysis focused on routes, as the locus of motion and manner information in graphic form. However, future work can better identify the nature of the event, length of a path, and types of manners (e.g., *twirling, spinning*). We might predict that, because S-languages collapse manner into the main verb, perhaps they depict more diverse and fine-grained manners than V-languages comics. Also, cues signaling manner itself (e.g., impact stars) and characteristics of motion lines (e.g., curly, or straight; multiple or a few) are indicative of the manner information such as several or longer lines reflecting faster actions (Hayashi et al. 2012). Such information will be annotated in future corpus analyses.

In addition, while our work focused on corpus analysis of motion events in visual narratives, experimental work could examine cross-cultural comprehension of these graphic motion events. Given our findings, we might ask whether motion lines affect the comprehension of motion events based on the spoken languages that people speak. Is the advantage of motion lines over postures found in motion comprehension (Friedman and Stevenson 1975) homogenous or modulated by linguistic typology? Might processing of (un)segmented motion events vary cross-culturally? Answers to these, among several possible questions, might also shed light on aforementioned concerns about using visual narratives as elicitation stimuli in verbal language typology studies.

Finally, our results raise further questions about the wider connections between graphic representations and linguistic typologies. Our findings suggest that the depiction of motion events across visual narrative sequences aligns with, and might be influenced by, the linguistic typologies of languages spoken by their authors. If substantiated further, this implies a permeability between expression in the verbal and graphic modalities similar to findings that linguistic representations might influence gestures (e.g., Özyürek et al. 2005). These results thus raise questions about what other aspects of linguistic typology might manifest in the structures of visual narratives and whether such influence is bidirectional, with representations from the graphic modality, in turn, influencing speech. Thus, while previous works have prevalently used visual narratives as tools to study the relationship between language and thought, they can provide valuable insights as objects of study in their own right.

## References

[j_cogsem-2022-2013_ref_001] Aske Jon (1989). Path predicates in English and Spanish: A closer look. ..

[j_cogsem-2022-2013_ref_002] Berman Ruth A., Slobin Dan I. (1994). *Relating events in narrative: A crosslinguistic developmental study*.

[j_cogsem-2022-2013_ref_003] Blomberg Johan (2014). *Motion in language and experience: Actual and non-actual motion in Swedish, French and Thai*.

[j_cogsem-2022-2013_ref_004] Brooks Penelope H. (1977). The role of action lines in children’s memory for pictures. *Journal of Experimental Child Psychology*.

[j_cogsem-2022-2013_ref_005] Carello Claudia, Rosenblum Lawrence, Grosofsky Alexis (1986). Static depiction of movement. *Perception*.

[j_cogsem-2022-2013_ref_006] Cohn Neil (2013). *The visual language of comics: Introduction to the structure and cognition of sequential images* (Bloomsbury Advances in Semiotics).

[j_cogsem-2022-2013_ref_007] Cohn Neil (2020). *Who understands comics?: Questioning the universality of visual language comprehension*.

[j_cogsem-2022-2013_ref_008] Cohn Neil, Cardoso Bruno, Klomberg Bien, Hacımusaoğlu Irmak (In prep). The Visual Language Research Corpus (VLRC): An annotated corpus of comics from Asia, Europe, and the United States. ..

[j_cogsem-2022-2013_ref_009] Cohn Neil, Maher Stephen (2015). The notion of the motion: The neurocognition of motion lines in visual narratives. *Brain Research*.

[j_cogsem-2022-2013_ref_010] Cutting James E. (2002). Representing motion in a static image: Constraints and parallels in art, science, and popular culture. *Perception*.

[j_cogsem-2022-2013_ref_011] Fortis Jean-Michel, Vittrant Alice (2016). Path-expressing constructions: Toward a typology. *STUF Language Typology and Universals*.

[j_cogsem-2022-2013_ref_012] Friedman Sarah L., Stevenson Marguerite B. (1975). Developmental changes in the understanding of implied motion in two-dimensional pictures. *Child Development*.

[j_cogsem-2022-2013_ref_013] Geisler Wilson S. (1999). Motion streaks provide a spatial code for motion direction. *Nature*.

[j_cogsem-2022-2013_ref_014] Hayashi Hiromasa, Matsuda Goh, Tamamiya Yoshiyuki, Hiraki Kazuo (2012). Visual effect of “speed lines” in manga an experimental study on spatial attention. *Cognitive Studies*.

[j_cogsem-2022-2013_ref_015] Ito Hiroyuki, Seno Takeharu, Yamanaka Miyuki (2010). Motion impressions enhanced by converging motion lines. *Perception*.

[j_cogsem-2022-2013_ref_016] Juricevic Igor (2017). Analysis of pictorial metaphors in comicbook art: Test of the LA-MOAD theory. *Journal of Graphic Novels and Comics*.

[j_cogsem-2022-2013_ref_017] Kawabe Takahiro, Miura Kayo (2006). Representation of dynamic events triggered by motion lines and static human postures. *Experimental Brain Research*.

[j_cogsem-2022-2013_ref_018] Kennedy John M., Ross Abraham S. (1975). Outline picture perception by the songe of paua. *Perception*.

[j_cogsem-2022-2013_ref_019] Kita Sotaro, Özyürek Aslı (2003). What does cross-linguistic variation in semantic coordination of speech and gesture reveal?: Evidence for an interface representation of spatial thinking and speaking. *Journal of Memory and Language*.

[j_cogsem-2022-2013_ref_020] Kourtzi Zoe, Kanwisher Nancy (2000). Activation in human MT/MST by static images with implied motion. *Journal of Cognitive Neuroscience*.

[j_cogsem-2022-2013_ref_021] Mayer Mercer (1969). *Frog, where are you?*.

[j_cogsem-2022-2013_ref_022] McCloud Scott (1993). *Understanding comics: The invisible art*.

[j_cogsem-2022-2013_ref_023] McCloud Scott (1996). Understanding manga. *Wizard Magazine*.

[j_cogsem-2022-2013_ref_024] Molés-Cases Teresa (2020a). On the translation of manner-of-motion in comics: Evidence from an inter- and intratypological corpus-based study. *Languages in Contrast*.

[j_cogsem-2022-2013_ref_025] Molés-Cases Teresa (2020b). Manner salience and translation: A case study based on a multilingual corpus of graphic novels. *Lebende Sprachen*.

[j_cogsem-2022-2013_ref_026] Naidu Viswanatha, Zlatev Jordan, Duggirala Vasanta, Van De Weijer Joost, Devylder Simon, Blomberg Johan (2018). Holistic spatial semantics and post-Talmian motion event typology: A case study of Thai and Telugu. *Cognitive Semiotics*.

[j_cogsem-2022-2013_ref_027] Özçalışkan Şeyda (2015). Ways of crossing a spatial boundary in typologically distinct languages. *Applied PsychoLinguistics*.

[j_cogsem-2022-2013_ref_028] Özyürek Aslı, Kita Sotaro, Allen Shanley, Furman Reyhan, Brown Amanda (2005). How does linguistic framing of events influence co-speech gestures?: Insights from crosslinguistic variations and similarities. *Gesture*.

[j_cogsem-2022-2013_ref_029] Regier Terry, Zheng Mingyu (2007). Attention to endpoints: A cross-linguistic constraint on spatial meaning. *Cognitive Science*.

[j_cogsem-2022-2013_ref_030] Slobin Dan I., Niemeier Susanne, Dirven René (2000). Verbalized events: A dynamic approach to linguistic relativity and determinism. *Evidence for linguistic relativity*.

[j_cogsem-2022-2013_ref_031] Slobin Dan I., Gentner Dedre, Goldin-Meadow Susan (2003). Language and thought online: Cognitive consequences of linguistic relativity. *Language in mind: Advances in the study of language and thought*.

[j_cogsem-2022-2013_ref_032] Slobin Dan I., Strömqvist Sven, Verhoeven Ludo (2004). The many ways to search for a frog: Linguistic typology and the expression of motion events. *Relating events in narrative: Typological and contextual perspectives*.

[j_cogsem-2022-2013_ref_033] Slobin Dan I., Maeder Costantino, Fischer Olga, Herlofsky William (2005). Linguistic representations of motion events: What is signifier and what is signified. *Outside-in, inside-out: Iconicity in language and literature 4*.

[j_cogsem-2022-2013_ref_034] Slobin Dan I., Hoiting Nini (1994). Reference to movement in spoken and signed languages: Typological considerations. ..

[j_cogsem-2022-2013_ref_035] Talmy Leonard, Shopen Timothy (1985). Lexicalization patterns: Semantic structure in lexical forms. Vol. III: Grammatical categories and the lexicon. *Language typology and syntactic description*.

[j_cogsem-2022-2013_ref_036] Tversky Barbara, Chow Tracy (2017). Language and culture in visual narratives. *Cognitive Semiotics*.

[j_cogsem-2022-2013_ref_037] Zlatev Jordan, Blomberg Johan, Devylder Simon, Naidu Viswanatha, van de Weijer Joost (2021). Motion event descriptions in Swedish, French, Thai and Telugu: A study in post-Talmian motion event typology. *Acta Linguistica Hafniensia*.

